# Knowledge, attitudes, and perceived barriers to seeking treatment for tuberculosis and diabetes in Ghana: a survey on world tuberculosis day

**DOI:** 10.3389/fpubh.2026.1826484

**Published:** 2026-06-18

**Authors:** Adwoa Asante-Poku, Michelle Yeboah-Manu, Stephen Osei-Wusu, Prince Asare, Augustine Asare Boadu, Emelia Konadu Danso, Phillip Tetteh, Ishaque Mintah Siam, Kenneth Mawuta Hayibor, Theophilus Afum, Emmanuel Afreh, Edmond Kwaku Ocloo, Isaac Darko Otchere, Nelly Arthur, Yaw Adusi-Poku, Charlotte-Alberta Cato, Abraham Adjei, Jane Afriyie-Mensah, Dorothy Yeboah-Manu

**Affiliations:** 1 Noguchi Memorial Institute for Medical Research, University of Ghana, Accra, Ghana; 2 Department of Research, University of Ghana Medical Center, Accra, Ghana; 3 Department of Chest Diseases, Korle-Bu Teaching Hospital, Accra, Ghana; 4 National Tuberculosis Control Programme, Ghana Health Service, Accra, Ghana; 5 Mamprobi Hospital, Mamprobi, Ghana

**Keywords:** diabetes mellitus, Greater Accra, knowledge, tuberculosis, world TB day

## Abstract

**Background:**

The global tuberculosis (TB) burden remains high, though several interventions have been instituted to fight the TB epidemic. Community perception and understanding play a critical role in health-seeking behaviors, ultimately influencing the acceptance and effectiveness of control efforts. In observance of World Tuberculosis (TB) Day in 2022 and 2023, we engaged the community and sought their understanding of TB and diabetes mellitus (DM).

**Methods:**

Interactive educational sessions were held in the outpatient department, basic schools, nursing college, and the market. A validated questionnaire was administered to consenting adults to evaluate their knowledge and views on TB and DM. Responses from participants were gathered using the KoboToolbox data collection program. Participants also underwent health screening, and those identified with TB and or DM were sent to Korle-Bu Teaching Hospital (KBTH) for appropriate management.

**Results:**

The survey documented responses from 285 individuals. The majority of the participants (63.5%) were women, aged 36–59 (38.3%), and had only basic school education (50.9%). Among the 179 individuals aware of TB, 48% accurately recognized bacteria or germs as its causative agents. Nonetheless, certain myths persisted, with around 6% attributing the disease to witchcraft or curses and 21% unclear of its genesis. Approximately 85% of individuals were aware of DM, while around 72% ascribed its etiology to dietary factors. Concerning community members’ engagement with patients diagnosed with TB, only 52.3% indicated they would have contact with those living with TB, whereas 82.5% expressed willingness to engage with those with diabetes mellitus. Approximately 55% of the participants perceived that both TB and DM are incurable.

**Conclusion:**

Our findings reveal critical knowledge gaps and widespread misconceptions about TB and DM. These gaps can be reduced through health education campaigns and community engagement that foster timely health-seeking behaviors, improving disease outcomes.

## Introduction

Tuberculosis (TB) is a disease of global health importance. It is a major contributor to antibiotic resistance and is the leading cause of mortality by a single infectious agent in the world. According to the Global TB 2025 report, TB is responsible for about 10.7 million incident cases and 1.08 million deaths in 2024 (1). Consequently, the World Health Organization (WHO) established the Global Plan to End TB to help eradicate TB as a public health threat by 2030 (2, 3). Despite significant global efforts, many countries, especially low- and middle-income countries (LMICs), are failing to meet key milestones in the WHO End TB Strategy (2).

Chronic and metabolic diseases such as human immunodeficiency virus (HIV) infection, malnutrition, cancer, and diabetes (DM) are critical barriers to achieving the WHO End TB Strategy targets, as they increase susceptibility to infection, accelerate progression to active disease, and diminish treatment success rate.

As its incidence rate rises rapidly, especially in LMICs with high TB rates, DM is a major issue. In 2016, the International Diabetes Federation (IDF) expected 422 million cases of diabetes mellitus (DM) worldwide, rising 60% to 642 million by 2040. This is problematic because these two disorders may create a bidirectional, harmful syndemic cycle that worsens both diseases’ prognoses.

Diabetes doubles or triples the chance of active TB, impairs the immune system, creates unusual clinical presentations, worsens disease severity, and lowers treatment outcomes. TB causes inflammation, which worsens insulin resistance, and some anti-TB drugs reduce the efficacy of oral antiglycemics, which worsens blood sugar control. Thus, effective TB/DM treatment requires coordination between TB and diabetes programs. The WHO and the International Union Against Tuberculosis and Lung Disease created the Collaborative Framework for Care and Control of TB and Diabetes in 2011 to address the link between the two diseases. However, a lack of planning and coordination makes execution challenging (4).

One reason for this is inadequate and inaccurate tuberculosis (TB) knowledge that is rife, particularly in LMICs (5). These misconceptions, coupled with resulting stigma, act as a hurdle to global TB control, as they cause unawareness of the significance of symptoms, postponed health-seeking behavior, delayed diagnosis, and poor treatment adherence. This creates a vicious cycle where stigma and misconception cause patients to hide their symptoms and avoid healthcare facilities, which increases the spread and burden of TB, further driving stigma against TB in society. Furthermore, inadequate information impairs medication adherence, thereby contributing to the emergence of TB strains resistant to available medications (6–8). The rise in DM cases, coupled with the relatively high rate of TB, now makes Africa an important region to study TBDM. Many African health facilities lack routine TB screening for DM due to expense, perceived complexity, and lack of treatment infrastructure (9). Standard diagnostic approaches have drawbacks for the majority of patients, leading to reliance on traditional medicine.

In Ghana, current knowledge in Ghana focuses on the clinical aspects of tuberculosis and diabetes mellitus separately. The combined condition’s community-level, human-centered features remain mostly unknown. People with TB with diabetes in developed nations, in particular, realize that DM triples the chance of acquiring active TB and that TB can affect blood sugar control. However, this is not a frequent public health message in Ghana. We do not know how much the general community is aware of the bidirectional link. Second, there is scant information about how communities explain a dual diagnosis. For example, if a patient develops tuberculosis after being diagnosed with diabetes, is it due to the diabetes itself, poor treatment adherence (self-blame), spiritual factors, or a separate “curse”? These narratives have a significant impact on care-seeking behaviors.

World Tuberculosis Day (WTBD), celebrated each year on 24 March, seeks to raise public awareness of the substantial health and socioeconomic impacts of TB, reduce stigma, promote political commitment, encourage research and innovation, highlight progress in TB control, recognize healthcare professionals, and empower the public to advance eradication initiatives. The day is notable for marking Dr. Robert Koch’s 1882 discovery of *Mycobacterium tuberculosis*. The themes for 2022 and 2023 WTBD, “Invest to End Tuberculosis and Save Lives” and “Yes! We can end TB!” respectively, aimed to foster optimism and advocate for improved leadership, increased funding, swift implementation of new WHO recommendations, adoption of innovations, expedited efforts, and cross-sector collaboration to combat the tuberculosis pandemic (10, 11).

While the co-epidemic of tuberculosis (TB) and diabetes mellitus (DM) poses a double danger to Ghana’s health system, community-level knowledge, attitudes, and perceived barriers to obtaining treatment for this comorbidity remain recognized (12, 13). Clinical studies have shown an increase in the prevalence of diabetes among tuberculosis patients. Primary research is scarce on patients’ knowledge, attitudes, and perceived barriers to seeking treatment for both diseases concurrently, especially in low- and middle-income countries (LMICs) like Ghana, where the dual disease burden is growing. Tuberculosis stigma and diabetes-related self-stigma are frequently researched independently, and the majority of the studies are conducted in clinical or residential settings. Existing assessments commonly assess knowledge and attitudes but rarely objectively quantify perceived restrictions (e.g., cost, location, stigma, time, and family obligations) as a separate barrier to seeking therapy rather than to receiving it. This study bridges a conceptual gap in health behavior research by distinguishing between restrictions and attitudes toward co-morbid infectious and non-communicable diseases. This study aimed to collect data during World Tuberculosis Day, a public awareness event, to identify a distinct, community-engaged demographic with different exposure to health messaging and treatment-seeking behaviors than those of usual clinic participants. No prior research has used a tuberculosis remembrance day to assess attitudes toward combined tuberculosis and diabetes (TB-DM) treatment.

This study, conducted on World Tuberculosis Day, takes advantage of a strategic public health opportunity to provide a cross-sectional snapshot of key community-level characteristics in Ghana to examine the amount of knowledge of the bidirectional link between tuberculosis and diabetes mellitus, including understanding of risk factors, transmission, and treatment interactions among Ghana community members.

The findings directly support progress toward numerous Sustainable Development Goal (SDG) targets (3.3, 3.4, and 3.8). This study bridges an evidence gap by linking community attitudes to SDG targets, enabling Ghana to meet its 2030 commitments for controlling infectious and non-communicable diseases.

To this end, the TB research team from the Noguchi Memorial Institute for Medical Research, University of Ghana (NMIMR-UG), in collaboration with the Ghana National TB Control Program (NTP), the Department of Chest Diseases of the Korle-Bu Teaching Hospital (KBTH), and the Mamprobi Hospital (MH), organized a TB awareness and screening exercise at some suburbs of Accra. These communities had previously been identified as a hotspot of TB transmission in the Accra Metropolis (14). We leveraged these two World TB Day celebrations to assess their knowledge, attitudes, and perceptions about TB and DM.

## Methodology

### Study design

This cross-sectional, community- and school-based study evaluates communities’ perceptions and attitudes toward diabetes and TB conducted during outreach activities. A cross-sectional study design was chosen because it provides a snapshot of a population, making it fast and efficient (15, 16). A 2-day TB awareness study was organized among the entire population of an upper primary school, a junior high school, public health nursing colleges, and two markets within the community during the two World TB Days. The participants were taken through the etiology of TB, clinical presentation, risk factors, and management, as outlined in Table 1. Afterward, participants who consented or assented to the study were asked questions to assess their knowledge, attitudes, and perceptions toward TB and DM. Second, the team organized a health screening exercise during the World TB Day celebrations, after which community residents who consented were once again enrolled into the study.

**Table 1:** Outline of activities for the awareness sessions.

Lecture 1	TB: Etiology, types, symptoms, and diagnosis	TB germ and types of TB TB tests TB symptoms and signs Diagnosis of TB
Lecture 2	TB: Transmission, infection control, and prevention	Spread of the TB germ Cough etiquette demonstration and respiratory waste management. BCG vaccination scar display
Lecture 3	TB: Risk factors, treatment, care, and support	TB risk factors, diabetes and HIV discussion TB treatment (directly observed treatment – short-course [DOTS]) TB stigma

### Study area

The Ablekuma South sub-Metropolitan District was chosen for this study. This sub-metropolitan district was chosen because previous studies identified it as a TB hotspot in the metropolitan area (17). An upper primary school, a junior high school, a public health nursing college, and two markets were randomly selected within this area as sites for the TB awareness workshops and health screening programs. Ablekuma South is the largest sub-metropolitan area in the Accra Metropolitan Assembly (AMA), covering 15.1 km^2^ with a population of over 257,000 people. The district includes many communities such as Korle Gonno, Mamprobi, Chorkor, and parts of the Korle-Bu area. The area is heavily involved in fishing and fishmongering. It also hosts several business entities, including banks, fuel stations, educational institutions, hotels, and two major markets (18). Schools provide an effective setting for the dissemination of preventive messages, particularly for populations with limited access to healthcare, which may be prioritized for interventions. Educating students promotes knowledge transfer to families and the broader community, potentially fostering lifelong healthy behaviors.

### Study population

A total of 285 people consented or assented to participate in the study.

#### Inclusion and exclusion criteria

Community members 11 years or older at the time of enrolment, residing in the designated district for a minimum of 6 months before data collection, and willing to participate with adequate mastery of at least one of the study languages (English, Twi, Ga, Ewe, or Dagbani) to comprehend the questionnaire and provide accurate responses were included in the study. The selected lower age limit aims to include early teens who may already encounter community health messages, familial sickness experiences, or initial diabetes risk factors (e.g., obesity and family history). No higher age restriction was imposed, contingent upon the individual’s ability to communicate effectively.

The timeframe guaranteed that participants received adequate exposure to local health services, cultural practices, and possible tuberculosis or diabetes health education initiatives. Transient residents (e.g., travelers and seasonal migrants residing for less than 6 months) were excluded to mitigate recall bias associated with local treatment-seeking behaviors.

Community members.

Participants who were acutely ill at the time of recruitment—such as those with fever (temperature >38.5 °C), respiratory distress, hemoptysis, or in a diabetic emergency (e.g., altered consciousness and severe dehydration)—Inability to provide consent or assent without a proxy, Individuals transiently present in the district (e.g., traders from other regions staying less than 1 week and tourists) and participants with prior participation in the same study were excluded from study.

#### Sampling technique

Consecutive sampling was selected as a conducive sampling technique.

#### Patient recruitment

Adolescents aged 11–17 were eligible to participate only after obtaining dual permission: written informed consent from a parent or legal guardian and written assent from the adolescent. Briefly, trained fieldworkers approached parents or legal guardians separately from the adolescent and explained the study objectives, voluntary participation, confidentiality, and the right to withdraw at any time without penalty. For guardians who could not read, fieldworkers read the entire consent form aloud in the local language (Twi, Ga, Ewe, or other local languages), and an impartial witness (not a study team member) attested to the informed consent process. Parents/guardians provided a signature or a thumbprint. After parental consent was documented, the adolescent was invited to a separate, age-appropriate assent discussion. The assent form used simplified language (at a reading level equivalent to ages 11–13) and explained the study in basic terms, emphasizing that participation was voluntary and that saying “no” would not affect their healthcare or standing in the community. Adolescents were asked to explain back what they understood (teach-back method) to confirm comprehension. Assent was documented by the adolescent’s signature (or a mark for those unable to write) on a separate assent form. For adolescents aged 11–14, a fieldworker also initialed a “witness to assent” line. No adolescent was enrolled without documented parental consent and documented adolescent assent. Data collection took place during the World TB Day celebration activities. Fieldworkers recorded a real-time track of unique participant identities (IDs) generated by combining district code, site code, and a sequential number, and verified each potential participant against this log.

### Step-by-step recruitment

Approach: Trained fieldworkers (public health nurses and research scientists, fluent in the dominant local language) wore visible study identification. They approached participants for engagement.

Screening and eligibility: Fieldworkers briefly explained the study and screened for inclusion criteria (age ≥ 11, resident ≥ 6 months, no cognitive impairment). For adolescents, they first identified whether a parent/guardian was present on site. If not, the adolescent was not recruited at that moment; instead, a flyer was given to the family.

Consent and enrolment: Eligible Adults were enrolled to be interviewed after providing written informed consent. For adolescents, the procedure described above (parental consent first, then adolescent assent) was followed. All consent and assent forms were administered in the participant’s preferred language. Fieldworkers recorded each consent/assent on a master log with a unique participant ID. Recruitment occurred after the official speeches to avoid coercion during high-profile sessions. Participants were offered a small bottle of water as a courtesy, not as an incentive to participate.

Questionnaire administration: The questionnaire was administered face-to-face in the local language by the same fieldworker who obtained consent/assent, lasting approximately 25–30 min. For participants with low literacy, fieldworkers read each item aloud and recorded responses. Adolescents were interviewed separately from their parents to minimize response bias.

### Data collection

A well-designed self-developed questionnaire was used to obtain data for the study. This questionnaire comprised multiple sections assessing participants’ knowledge of the etiology, route of transmission, clinical presentation, and management of tuberculosis. Additionally, the study probed into the participants’ attitudes and perceptions toward both TB and DM. Responses from participants were collected using the KoboToolbox data collection program.

### Quality control and data management

Data was collected using KoboCollect. Data collectors received training on the study protocols, ethical considerations, and proper use of the tool to ensure consistency and accuracy in data entry. The tool incorporated validation checks such as required fields, skip patterns, and range limits to minimize errors at the point of collection.

Data was stored on a password-protected device and securely uploaded to a protected server with restricted access to ensure data security. Missing data were handled systematically: records with substantial missing information were excluded, while variables with minimal missingness were analyzed using available case analysis.

### Data analysis

Descriptive analysis was carried out using Stata version 17. The dataset was checked for completeness, and no missing observations were found. Had there been missing values on key variables, those observations would have been excluded from the analysis to maintain the integrity of the results. Descriptive statistics were utilized to summarize all variables. Frequency distributions were used to calculate proportions for all categorical variables. The mean age and standard deviations were computed to assess how closely the individual data values matched the mean. Twelve knowledge-related questions were designed to assess the study respondents’ knowledge level. Responses were dichotomously coded: a value “1” was assigned for correct responses and “0” for incorrect or “do not know” responses. The researcher made this determination to reflect an accurate understanding of the key concepts. A composite score was then computed based on the code assignment, and a respondent was classified as having good knowledge if their scores were average or above on the questions. Attitudes toward people living with leprosy, BU, and yaws were categorized into poor and good based on scores from the nine questions regarding the diseases. Respondents who scored less than the average (5) were rated as having a “poor attitude,” while those who scored 5 or higher were rated as having a “good attitude.” The main outcome variables of interest included knowledge, attitude, healthcare-seeking behavior, and stigma.

### Ethical review

Ethical approval for the study was obtained from the NMIMR ethical committee (NMIMR-IRB-NMIMR-IRB CPN 077/23–34 revd. 2025). Informed consent was obtained from all participants after the study’s purpose was explained to them. Minors were also asked for their assent after their parents consented to the study. To protect participant confidentiality and privacy, names and other identifying characteristics were excluded from the data entry.

## Results

### Sociodemographic characteristics of the participants

Table 2 shows the sociodemographic profiles of the 285 individuals who participated in this study. The majority of the participants were female (63.5%), aged 36–59 (38.3%), and had complete and basic education (50.9%). Participants were also mostly unmarried (single: 49%; divorced: 7.4%; and widowed: 0.8%). Regarding education, the majority of participants (*n* = 98; 34.4%) were unskilled laborers, with students making up the next-largest group at 30.9% (88). Christians (84.7%) and members of the Ga/Adangbe tribe (46.7%) comprised the majority of participants.

**Table 2:** Sociodemographic characteristics of the participants.

Demographics	Frequency (%)
Age group	
10–17	62 (21.8)
18–35	72 (25.3)
36–59	109 (38.2)
>60	42 (14.7)
Sex	
Male	104 (36.5)
Female	181 (63.5)
Level of education	
No education	36 (12.6)
Basic education	145 (50.9)
Secondary education	68 (23.9)
Tertiary/graduate	36 (12.6)
Occupation	
Unemployed	30 (10.5)
Student	88 (30.9)
Casual laborer/unskilled laborer	98 (34.4)
Trader	47 (16.5)
Skilled laborer/artisans	6 (2.1)
Retired/pension	16 (5.6)
Religion	
Christian	241 (84.7)
Islam	34 (11.9)
African traditional	2 (0.7)
Other specify	8 (2.7)
Ethnic group	
Akan/Twi/Fante	82 (28.7)
Ga/Adangbe	113 (46.7)
Ewe	10 (3.5)
Guan	3 (1.1)
Northerner	29 (10.2)
Others	28 (9.8)

### Knowledge of TB and DM

The media (audio/print) emerged as the major source of information for both TB (47.5%) and DM (73.3%). Regarding to TB, community members were also identified as an important source of information (43.5%). Less than half (48%) of the participants identified bacteria as the cause of TB. When probed about the clinical symptoms for more than 2 weeks, as a symptom of TB. Moreover, 50.5% knew that TB is transmitted through the air when an infected person coughs or sneezes, and 48.1% mentioned that a cough lasting for 2 weeks or more is a major sign and symptom of TB. As to the seriousness of TB disease, 54.7% of participants pointed out that the disease is life-threatening. 81.7% of the respondents indicated TB can be prevented by covering the mouth and nose when coughing. However, a minority of the respondents indicated that TB can be prevented by avoiding eating raw meat (Table 3). The majority, 55.4%, indicated that TB is curable, and a greater number of the participants (66.7%) knew that anybody can be infected with TB disease. Additionally, 55.4% of the respondents were aware of DOTS, with a majority indicating that the drugs are available and free for the treatment of TB.

**Table 3:** Community members’ knowledge of tuberculosis and diabetes.

Variable	Frequency (%)
Tuberculosis	
How did you become aware of tuberculosis?	
I was/am a patient	3 (1.7)
I know someone who had it	53 (29.6)
Community members talk about it	81 (45.3)
Media (radio/TV/newspapers/social media)	85 (47.5)
From Hospital staff/Posters	41 (22.9)
What are the causes of tuberculosis?	
Bacteria/germs	86 (48.0)
Witchcraft/spirits/curse	11 (6.2)
Do not know	38 (21.2)
What is the mode of transmission for tuberculosis?	
Through the air when an infected person coughs/sneezes	144 (50.5)
Living with a TB patient	34 (11.9)
What are the signs and symptoms of tuberculosis?	
Cough lasts 2 weeks +	137 (48.1)
Night sweats	31 (10.9)
Bloody sputum	44 (15.4)
Chest pains	49 (17.2)
Fatigue	31 (10.9)
Shortness of breath	16 (5.6)
What is your assessment of the severity of tuberculosis?	
Life-threatening	156 (54.7)
Not very serious	109 (38.2)
Do not Know	14 (4.9)
Are you aware of any treatment for tuberculosis?	
Yes	158 (55.4)
No	113 (39.6)
Do not know	14 (4.9)
What do you think is the expense of tuberculosis therapy	
It is free	156 (54.7)
Pay some fee	80 (28.1)
Do not know	49 (17.2)
What do you think is the best way for tuberculosis prevention	
Covering of the mouth when coughing	232 (81.4)
Avoid eating meat	53 (18.6)
Who can contract tuberculosis?	
Anybody	190 (66.7)
Children	70 (24.6)
Adults (60 years and above)	25 (8.8)
Diabetes	
What is your source of knowledge regarding diabetes?	
I was/am a patient	6 (2.1)
I know someone who had it	145 (50.9)
Community Members talk about it	79 (27.7)
Radio/TV/Newspapers	209 (73.3)
From Hospital staff/Posters	85 (29.8)
How does someone obtain diabetes?	
Hereditary (family disease)	8 (2.8)
Dietary	169 (59.3)
Insulin resistance	3 (1.1)
No Idea	17 (6.0)
What are the signs and Symptoms of diabetes?	
Obesity	218 (90.5)
Increased thirst	206 (85.5)
Frequent urination	180 (74.7)
Extreme hunger	223 (92.5)
Unintentional weight loss	191 (79.3)

Regarding community members’ knowledge of DM, 2.8% reported that one can get DM through hereditary means, while 59.3 and 1.1% reported it through diet and insulin resistance, respectively. 6.0% of patients, however, had no idea about the cause of DM. The majority of the patients (92.5%) selected extreme hunger as a sign/symptom of diabetes. The majority, 195 (68.4%), mentioned that DM can be life-threatening. 158 (68.4%) agreed that there is a cure for diabetes, while 38 (13.3%) are unsure whether DM has a cure. Finally, the majority of participants (*n* = 158; 55.4%) indicated that DM should be treated at the hospital; however, a significant number (*n* = 50; 17.5%) were unsure where DM could be treated.

### Attitudes and perceptions toward TB and DM

**Figure 1:**
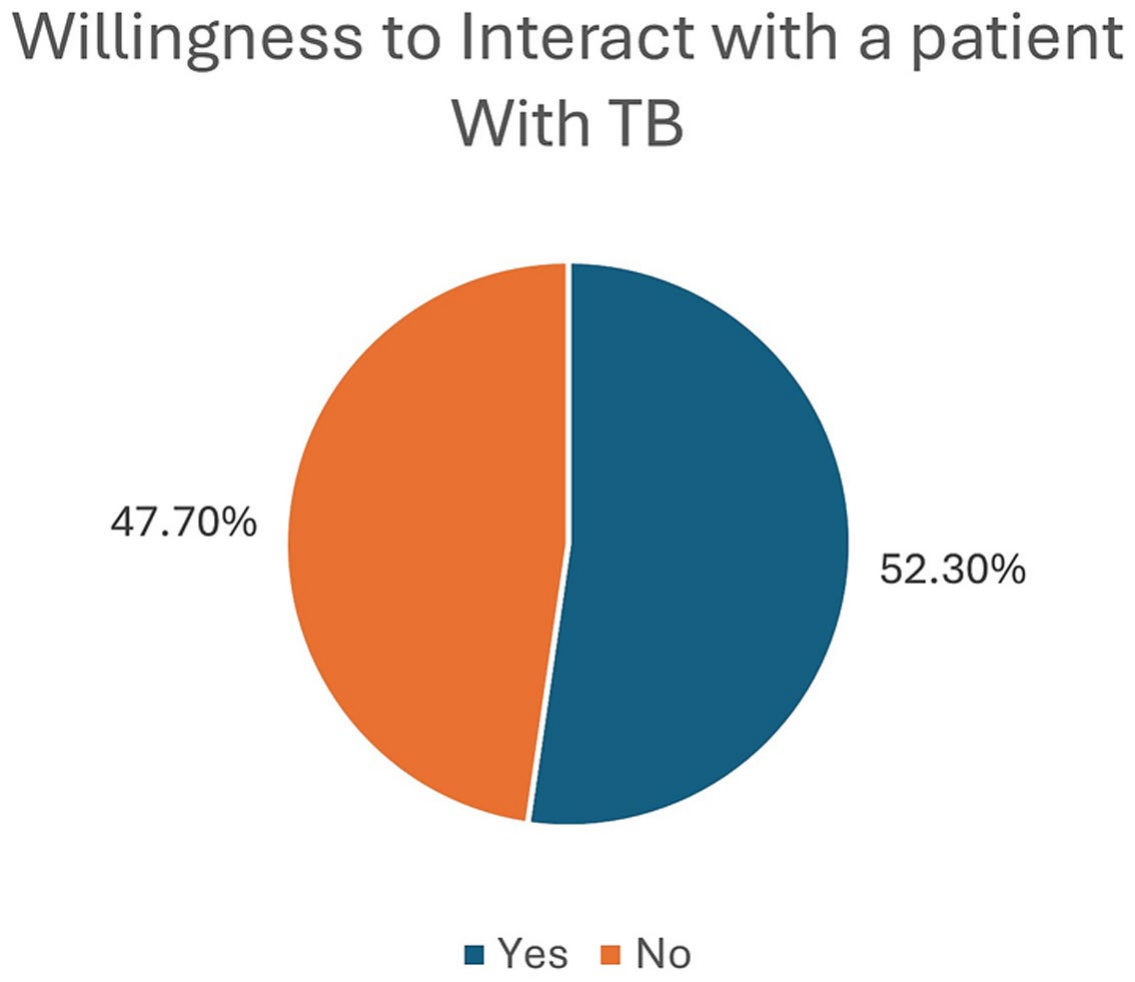
Willingness to interact with a patient with TB.

**Figure 2:**
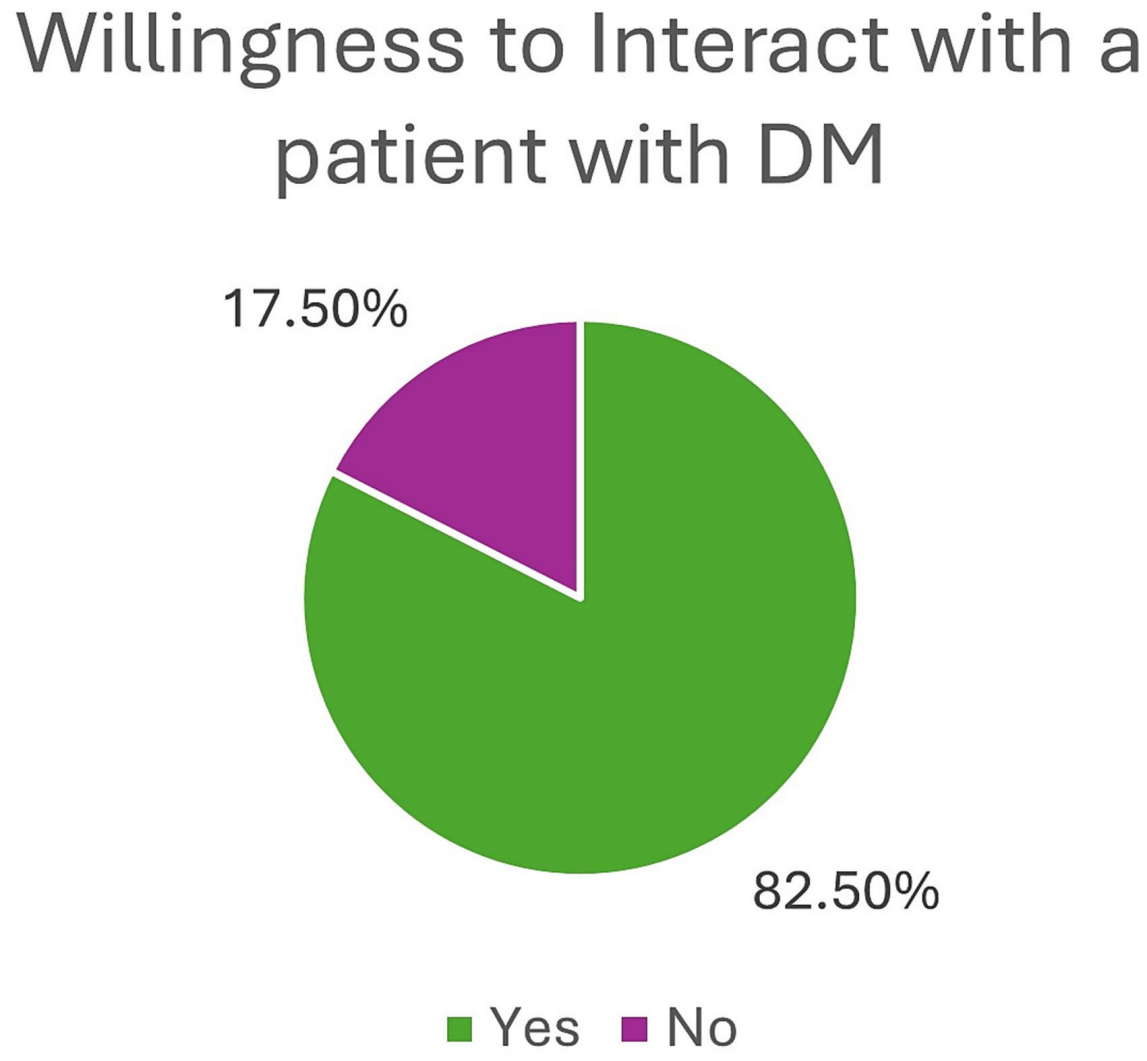
Willingness to interact with a patient with DM.

**Figure 3:**
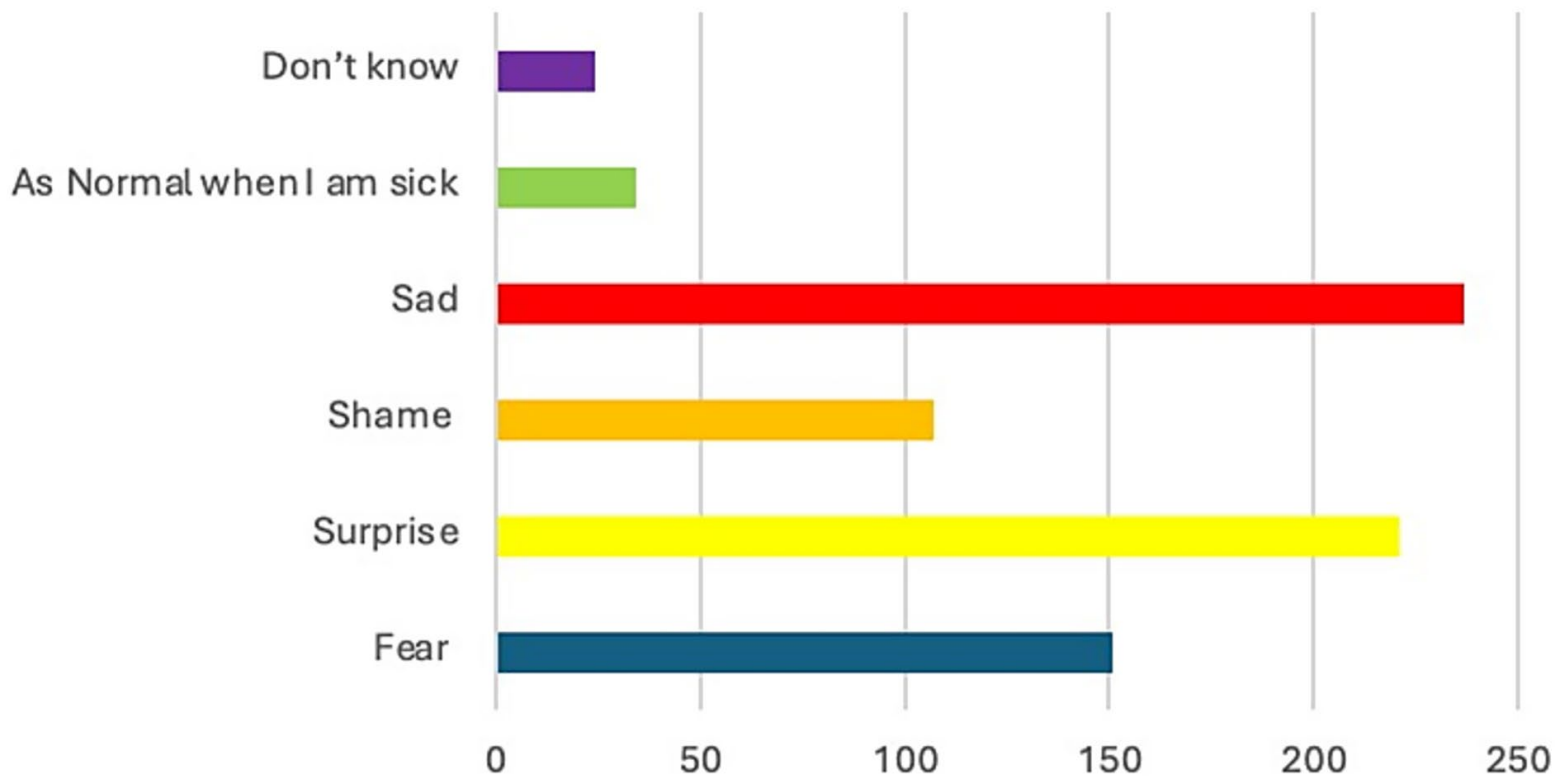
Reactions from contracting TB.

**Figure 4:**
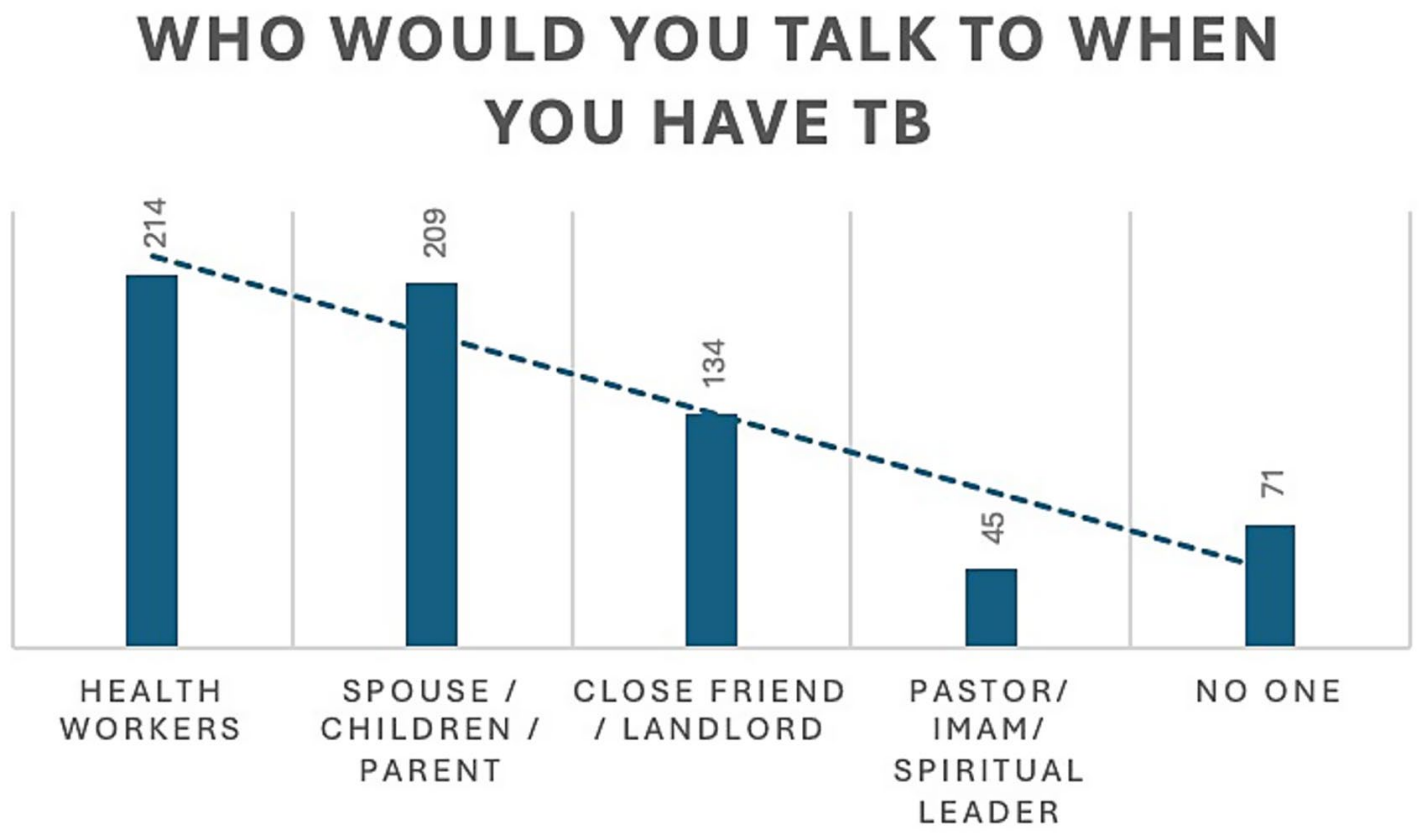
The preferred persons to talk to when participants find out they have TB.

Regarding community members’ engagement with TB patients, only 52.3% indicated they would be willing to have contact with people living with TB (Figure 1), whereas 82.5% expressed willingness to have contact with individuals with DM (Figure 2). Concerning the emotional responses of community members upon discovering they have TB, 53% indicated they would feel scared, 77.5% expressed surprise, and 83.2% reported feeling sad (Figure 3). Furthermore, 75% of community members indicated willingness to consult a health worker, while 73.3% expressed readiness to communicate with their spouse, children, or parents. A majority (81.8%) of the participants indicated that they would seek medical attention promptly upon experiencing TB symptoms (Figure 4).

### Barriers to tuberculosis and diabetes treatment-seeking

**Figure 5:**
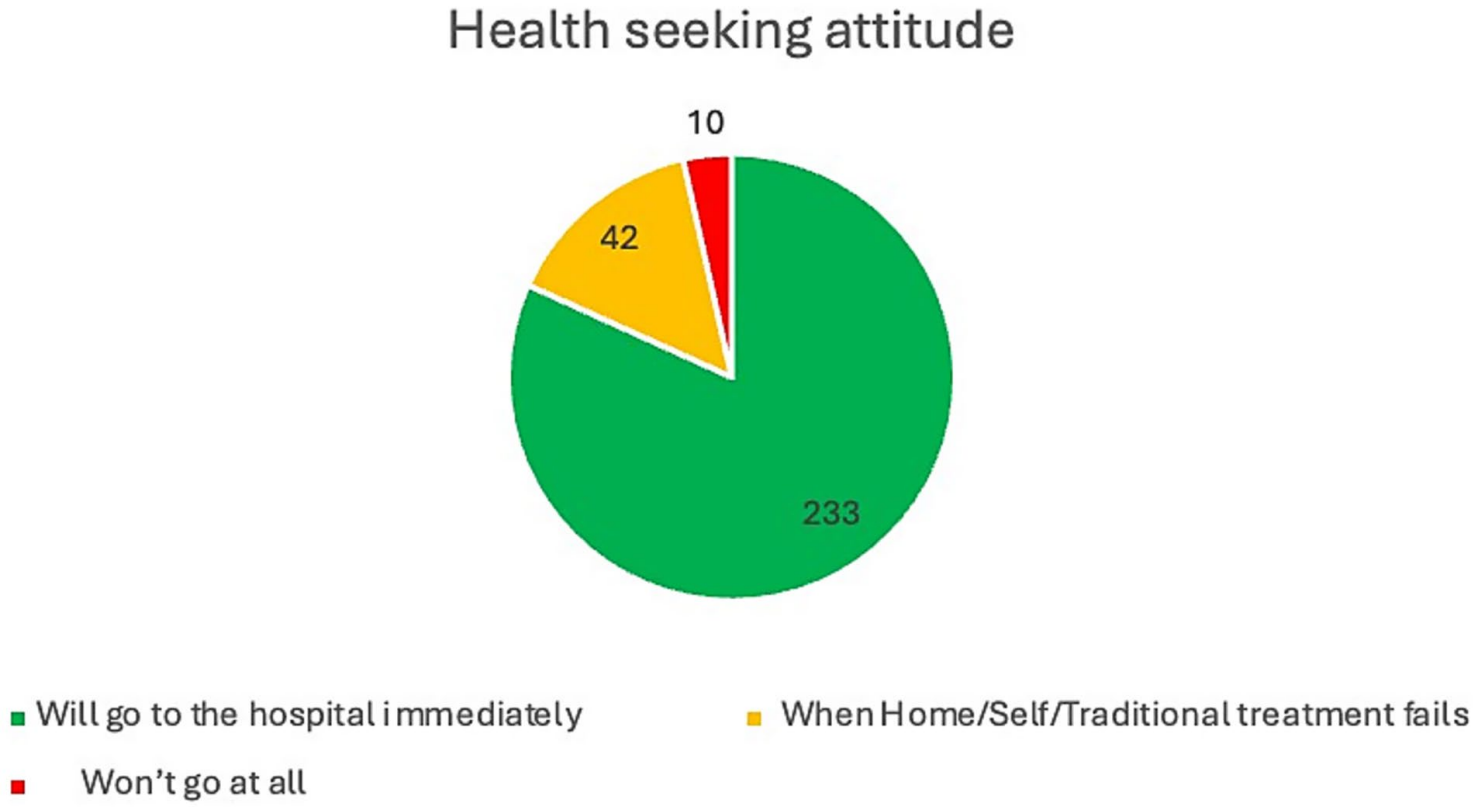
Health-seeking attitude for TB and diabetes.

The study also explored the threshold at which participants would seek assistance from a health center upon recognizing symptoms of TB or DM. The majority of the respondents (81.7%) indicated that they only go to a health facility after home treatment or traditional treatment failed if they recognize symptoms of TB or DM (Figure 5). The participants were also asked about barriers that prevent them from seeking treatment for TB and DM, and 66% of the respondents cited elevated treatment expenses as the reason for their inability to access healthcare. At the same time, 11.6% attributed it to the considerable distance from their homes to health facilities. When asked what influenced their choice of traditional/faith/herbal healers over orthodox medicine, 68.4% of participants indicated that cost was a factor, while 10.5% of them mentioned that receiving treatment from the former is less stigmatizing than from the latter. Finally, the majority of patients (49.8%) admitted that there was enough information about TB available in the community. Similarly, 48.1% of respondents indicated that there was enough information on DM available in the community (Table 4).

**Table 4:** Perceived limitations toward treatment-seeking for TB and DM.

Variable	Frequency (%)
What barriers prevent people with tuberculosis and diabetes mellitus from accessing healthcare services?	
Treatment cost	188 (66.0)
Distance to health facility	33 (11.6)
Transportation costs	63 (22.1)
Bad attitude of the health personnel	22 (7.7)
Poor quality of care	27 (9.8)
What factors do you think cause patients with tuberculosis or diabetes mellitus to choose therapy from traditional healers, herbal practitioners, or faith healers?	
Treatment costs	195 (68.4)
Transportation cost	6 (16.1)
Proximity	27 (9.8)
Quality of care	75 (26.3)
Less stigmatizing	30 (10.5)
Education on TB is limited	
Yes	124 (45.3)
No	137 (49.8)
I do not know	19 (6.7)
Education on DM is limited	
Yes	129 (45.2)
No	137 (48.1)
I do not know	19 (6.7)

## Discussion

Our study provides insights into the knowledge, attitudes, and perceived limitations affecting the treatment-seeking behavior for TB and DM in Ghana, particularly within suburban communities. Overall, the results obtained reveal that participants generally have an idea of TB and DM; however, many misconceptions remain. This is unsurprising as the majority of the participants had at least a basic education. This is consistent with other studies that found some form of formal education (9, 19–23).

The primary source of information for TB and DM was identified as the media, aligning with previous studies that found a significant influence of mass media on public awareness (24–26). This finding emphasizes the necessity of utilizing community frameworks and mass communication platforms to disseminate accurate health information. Community members also serve as a major source of information on TB, proving that TB champions and volunteers are vital in the country’s efforts to combat the disease through education, awareness, and active case finding.

Almost half (48%) of the participants accurately identified bacteria as the cause of TB. Additionally, a considerable proportion had the correct knowledge of symptoms of TB, mode of transmission of TB, prevention of TB, and the fact that anyone could contract TB. A little over half of the participants also knew that TB could be life-threatening; however, it could be cured, and treatment was free. This outcome could probably be attributed to the numerous public health education efforts in the sub-metropolitan areas. Ablekuma South has hosted and continues to host numerous TB research studies. Moreover, the Korle-Bu Teaching Hospital, the country’s largest hospital with a robust chest clinic, is in this region. As a result, the sub-metropolitan areas are periodically exposed to public health education on TB. However, despite many educational efforts in the district, a significant number still identified witchcraft/spirits/curses as the cause of TB, while the rest indicated they did not know. In addition, more than 40% of participants did not realize that TB was serious, had a free cure, and 18.6% believed avoiding meat could prevent TB. This is consistent with a previous study that determined that there are persistent cultural beliefs among some populations in Ghana regarding TB (27). It suffices that campaigns about TB should focus more on addressing cultural logics or beliefs associated with its causation, prevention, and management.

Similarly, awareness of DM was found to be limited, which is consistent with a study conducted in a different region of Ghana (28). About 60% of participants recognized dietary factors as a cause of DM, while very few people recognized hereditary and insulin resistance as causes of DM. This highlights a significant gap in public health awareness of the disease’s pathophysiology. This disparity can lead to delays in diagnosis and improper management, such as focusing solely on diet while neglecting exercise and medication adherence, leading to sub-optimal outcomes. It is also important to note that more than half of the participants (55%) believed that DM can be cured. Although research indicates that type 2 diabetes can be reversed or put into remission via rigorous lifestyle modifications, the current medical consensus maintains that there is no permanent treatment for diabetes mellitus. There is currently no cure for type 1 diabetes.

This suggests that healthcare providers need to emphasize this when educating patients and the public about DM, as it affects treatment compliance and outcomes.

With regard to attitudes toward TB and DM, stigma continues to be a major obstacle to the management of both diseases, though not equally. TB patients face more stigma than DM patients. Only 52.3% of participants indicated their willingness to interact with TB patients, in contrast to 82.5% who felt at ease engaging with individuals diagnosed with DM. This syncs with a study performed in South India that estimated that patients with TB face about 8 times the stigma as compared to patients with DM (29). This intense stigma may prevent TB patients from seeking early diagnosis and treatment, thereby increasing the risk of transmission and the development of drug-resistant strains. This suggests that TB management should be integrated across all hospitals nationwide to transform TB from an isolated, mysterious disease into a routine component of healthcare, thereby reducing stigma (30, 31). Additionally, the emotional reactions reported by participants, such as fear (53%), sadness (83.2%), and surprise (77.5%), in response to a hypothetical TB diagnosis. It suffices that a diagnosis of TB may evoke feelings of shame, guilt, and social withdrawal, hence the need for the inclusion of psychosocial supports in TB care or treatment (32).

The majority of participants (81.8%) indicated a willingness to seek medical care when exhibiting symptoms of TB or DM, which aligns with findings from other studies (22, 33, 34). Nevertheless, the gap between expressed willingness and actual healthcare-seeking actions implies that external factors, including financial constraints and access to healthcare services, may impede prompt responses (12). While the National TB Control Program fully covers TB treatment, DM is partially covered under the National Health Insurance Scheme. Indirect expenses, such as transportation, lost work time, and diagnostic fees, can deter people from seeking prompt medical attention. This aligns with other studies that hidden costs associated with TB diagnosis and treatment lead to delays in accessing healthcare (35, 36).

A particularly alarming finding is that 68% of participants preferred to consult traditional healers before seeking care at a healthcare facility. This lends credence to other studies that traditional healing remains a crucial care option for people in developing nations due to their cultural beliefs and limited access to formal healthcare services (37–39). It is important to note that although traditional healers are essential in many communities, delays in receiving biomedical treatment can exacerbate outcomes for TB and DM. Therefore, enhancing collaboration between healthcare professionals and traditional healers through integrated health education initiatives could promote earlier diagnosis and improve treatment adherence (40).

### Strengths of the study

This study is one of the first studies to explore the public’s knowledge, attitudes, and practices (KAP) regarding TB and DM. This is important due to the syndemic nature of TB and DM, especially in the face of the rapidly increasing burden of diabetes worldwide. Another key strength of this study is its adoption of a community-based approach and inclusion of multiple settings.

### Limitations of the study

The key limitations of this study include selection bias through the use of a consecutive sampling technique, a lack of inferential analysis, potential response bias, and limited generalizability. In addition, our study did not further explore the community’s KAP regarding the relationship between DM and TB, as well as TB/DM comorbidity.

### Policy and practice implications

We maintain that addressing these issues requires instituting a holistic strategy that encompasses targeted educational initiatives and stigma-reduction efforts to improve the community’s knowledge of TB and DM.

This World Tuberculosis Day survey reveals the potential to improve integrated tuberculosis and diabetes (TB-DM) care in Ghana. This study goes beyond clinical prevalence estimates to reveal information gaps, stigmatizing attitudes, and modifiable perceived barriers to treatment seeking, informing effective policy, frontline practice, community engagement, and future research.

Current Ghanaian health policies target tuberculosis and diabetes through separate vertical programs, but our data reveal that people view both diseases as interconnected barriers to care. As a result, policymakers should incorporate TB-DM screening and referral pathways into the National Health Insurance Scheme (NHIS) benefit package, with symptom screening and point-of-care glucose testing at the same visit. Furthermore, during routine visits, frontline health personnel should employ a bidirectional educational approach. This includes explaining to a tuberculosis patient how uncontrolled diabetes might delay sputum conversion and increase the risk of relapse. Diabetes patients should be educated about their elevated tuberculosis risk and the significance of obtaining immediate medical assistance if they develop a persistent cough. Visual job aids (e.g., flip charts demonstrating the “two-way street” between TB and DM) and teach-back methods can help to assure understanding. These educational sessions should take place not only in tuberculosis clinics, but also in antenatal care, HIV clinics, and outpatient departments, where undiagnosed comorbid patients may first arrive.

Furthermore, facility-based therapies should involve counseling protocols (e.g., “Many people with TB also have high blood sugar; this is not a personal failing”). Peer support groups for people with simultaneous TB-DM provide a secure area to share coping strategies and reduce internalized stigma. Our World Tuberculosis Day survey platform successfully engaged general audiences. Future events should feature testimonials from recovered TB-DM patients, as well as an invitation for community leaders to freely express their own chronic disease experiences, to destigmatize both conditions.

The excellent recruitment of participants on World Tuberculosis Day implies that disease-specific memorial days offer untapped opportunities for integrated screening. We recommend that district health management teams set up combined TB-DM screening kiosks at markets, truck stops, and churches during these events, offering simultaneous symptom screening, random blood glucose readings, and immediate referrals. Community health volunteers (e.g., Community-based Health Planning and Services [CHPS] compound workers) should be trained to use simple checklists—“Have you had a cough for 2 weeks?” and “Have you been very thirsty or losing weight without trying?”—to initiate referrals for both conditions. To overcome perceived limitations such as transportation costs and time away from work, screening should be conducted in conjunction with current social assistance programs (e.g., LEAP cash transfer distribution locations) and in the early morning or on weekends.

Future interventional research. This survey provides a baseline for high-priority research initiatives. Cluster-randomized controlled trials assessing the effects of integrated TB-DM education packages (e.g., group counseling plus take-home pamphlets vs. standard care) on knowledge, attitudes, and treatment-seeking behavior after 6–12 months. Implementation science research is required to determine the feasibility and cost-effectiveness of incorporating bidirectional screening into primary care. Screening yield, patient retention, and linkage to care are all possible outcomes. Third, a qualitative longitudinal study should follow people who reported severe perceived limits but did not seek treatment, looking at how these obstacles interact dynamically over time (for example, whether a single financial shock delays treatment). To determine the generalizability of our findings, future studies should compare event-based and clinic-based populations, as our sample was drawn from a single awareness day. Mixed-methods intervention designs could evaluate changes in self-reported attitudes and treatment initiation rates using stigma-reduction toolkits (for example, motivational interviewing for health personnel and a community-wide radio broadcast). These findings have the potential to help scale combination TB-DM care in Ghana and other low- and middle-income countries.

## Conclusion

Our findings reveal notable deficiencies in knowledge, attitudes, and perceived obstacles regarding TB and DM among urban communities in Ghana. Although residents demonstrate a readiness to pursue medical assistance, challenges such as financial limitations, social stigma, and dependence on traditional medicine hinder effective management of these diseases.

## Data Availability

The original contributions presented in the study are included in the article/supplementary material, further inquiries can be directed to the corresponding authors.
